# Mass and Charge Transfer in a Polymeric NiSalen Complex at Subzero Temperatures

**DOI:** 10.3390/polym15051323

**Published:** 2023-03-06

**Authors:** Elena V. Alekseeva, Julia V. Novoselova, Dmitrii V. Anischenko, Vasiliy V. Potapenkov, Oleg V. Levin

**Affiliations:** Institute of Chemistry, Saint Petersburg University, 7/9 Universitetskaya nab., 199034 St. Petersburg, Russia

**Keywords:** metal-salen-type polymers, cyclic voltammetry, low-temperature electrode material, impedance spectroscopy

## Abstract

Electrochemical energy storage systems have a wide range of commercial applications. They keep energy and power even at temperatures up to +60 °C. However, the capacity and power of such energy storage systems reduce sharply at negative temperatures due to the difficulty of counterion injection into the electrode material. The application of organic electrode materials based on salen-type polymers is a prospective approach to the development of materials for low-temperature energy sources. Poly[Ni(CH_3_Salen)]–based electrode materials synthesized from different electrolytes were investigated by cyclic voltammetry, electrochemical impedance spectroscopy and quartz crystal microgravimetry at temperatures from −40 °C to 20 °C. By analyzing data obtained in various electrolyte solutions, it was shown that at subzero temperatures, the process of injection into the polymer film, together with slow diffusion within the film, predominantly limit the electrochemical performance of electrode materials based on poly[Ni(CH_3_Salen)]. It was shown that the deposition of the polymer from solutions with larger cations allow the enhancement of the charge transfer due to the formation of porous structures facilitating the counter-ion diffusion.

## 1. Introduction

Commercial application of electrochemical energy storage devices (EESD), batteries and supercapacitors at low temperatures is limited by the sharp decrease in their capacity and power [1,2]. This problem could be solved using several methods. One of them is the addition of various additives to the electrolyte [3,4,5]. Another approach is the use of an external or internal heating system [6,7]. Using such systems increases the cost, weight, and startup time of the device, as well as reduces its stability, efficiency and cycling life [2].

The main reason for the decrease in electrode efficiency at low temperatures is the slow lithium-ion transfer process into the electrode material [8]. Lithium, as well as other charge-compensating ions, exists in the electrolyte solution in the solvated form. Therefore, ion transfer, which is necessary to maintain electroneutrality of the electrode, consists of three stages: transport of the solvated ion to the electrode/electrolyte interface, desolvation of the ion and the intercalation of the ion through the interface. The rate of the desolvation stage drastically decreases at subzero temperatures, while in the case of inorganic electrode materials, solvated ion injection could lead to damaging material due to the rigidity and low interlayer distance of the crystal lattices and the presence of solid electrolyte interphase (SEI) layers on their surfaces [8,9].

The flexible, porous structure of organic cathode materials provides an efficient process of intercalation–deintercalation of charge-compensating counterions even at low temperatures [2]. Such materials may be able to use both solvated and desolvated ions to maintain electroneutrality [10,11,12]; however, we could not find any relevant discussion on the kinetics of competing processes of insertion of solvated and nonsolvated ions and their influence on the electrochemical performance of the material. Polymers based on nickel–salen-type complexes are promising materials for cathodes of organic batteries and supercapacitors, which work effectively at low temperatures. However, some aspects of the low-temperature charge-transfer kinetics in such organic electrode materials remain unclear. For example, the rate-determining step and its temperature dependence have not been studied in detail for those polymers. It is known that accounting for the effect of counterion solvation is critical for predicting the electrochemical activity of materials at negative temperatures [13]. We believe exploring such dependences is necessary to improve the understanding of organic cathode properties and their range of applicability. This, in turn, will help in the future realization of organic electrode materials with superior characteristics for low-temperature electrochemical energy storage. To achieve this goal, in this work, films of the poly[Ni(CH_3_Salen)] complex with different porosity were studied using various electrochemical and physicochemical methods.

## 2. Materials and Methods

**Figure 1:**
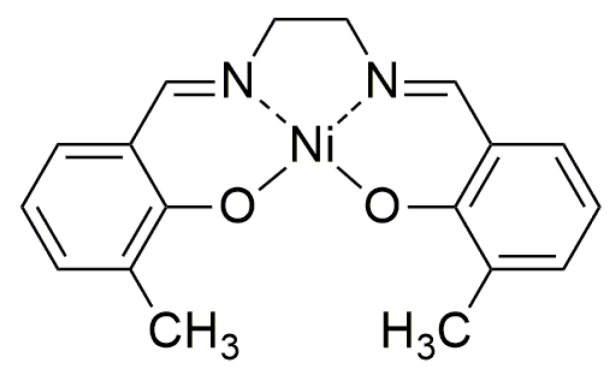
Structure of monomeric complex [Ni(CH_3_Salen)].

Monomeric complex [Ni(CH_3_Salen)] (Figure 1) was synthesized according to the protocol described elsewhere [14]. [Ni(CH_3_Salen)] was polymerized by applying a constant potential of 1 V to the working glassy carbon disk electrode (surface area is 0.07 cm^2^) immersed in acetonitrile (AN) containing 10^−3^ M of monomer and one of the salts from the following list: tetramethylammonium tetrafluoroborate (Me_4_NBF_4_) (Aldrich), tetrabutylammonium tetrafluoroborate (Bu_4_NBF_4_) (Aldrich) or tetrabutylammonium bis(trifluoromethanesulfonyl)imide (Bu_4_NTFSI) (Aldrich). Charge of the polymerization was 0.001 C.

Polymerization of monomers and all electrochemical measurements were carried out in a three-electrode cell consisting of a working electrode, a counter electrode (platinum plate, 1 cm^2^) and a non-aqueous Ag/Ag^+^ reference electrode (Ag wire immersed in acetonitrile containing 0.1 M AgNO_3_, voltage was 0.3 V vs. aqueous Ag/AgCl (KCl sat.) reference electrode).

Electropolymerization was carried out on Metrohm Autolab PGSTAT12 and Metrohm Autolab PGSTAT302N (Eco-Chemie, Utrecht, The Netherlands).

The polymers were studied using cyclic voltammetry (CV) in anhydrous solutions of Me_4_NBF_4_ (0.1 M)/AN, Bu_4_NBF_4_ (0.1 M)/AN or Bu_4_NTFSI (0.1 M)/AN in the potential range of −0.3 to −1 V. The polymer-modified electrodes were cycled with scan rates from 1 mV s^−1^ to 2 V s^−1^ during 3 cycles at the following temperatures: 20, 0, −20 and −40 °C. Temperature control was performed using a LOIP cryostat (model FT-311-80, St. Petersburg, Russia) with the temperature probe immersed in the electrolyte. Set temperature of an experiment was achieved in 3 to 5 h depending on the set temperature and the ambient temperature. Then, the working solution was acclimated for 1 h at the set temperature. The third cycle of each CV is presented and discussed in this manuscript.

All electrochemical experiments were performed on Metrohm Autolab PGSTAT12 and Metrohm Autolab PGSTAT302N (Eco-Chemie, The Netherlands). In each measurement, the automatic IR compensation was calibrated and turned on to eliminate the effects of the resistance of the solution.

The polymer mass was calculated according to Faraday’s law:

(1) $$m_{theor}=\frac{M_{r}\cdot Q_{poly}}{Fn_{poly}},$$

where *M_r_* is the molecular weight of the monomer fragment, g mol^−1^; *Q_poly_* is the polymerization charge, C and *n_poly_* = 2 is the number of electrons spent on polymerization [14].

Specific capacitance (*C_s_*) of the polymer was calculated based on cyclic voltammetry data using Equation (2), corresponding to [15], as shown here:

(2) $$C_{S}=\frac{\int i_{C}dt}{x\Delta E},$$

where *i_c_* is the cathodic current, Δ*E* is the potential range of cyclic voltammograms and *x* is the mass of the electroactive material deposited on the working electrode or the area of the working electrode in the case of the calculation of gravimetric capacitance or areal capacitance, respectively.

An EQCM test was performed on a QCM200 quartz crystal microbalance analog controller and a QCM25 Crystal Oscillator (Stanford Research Systems, Sunnyvale, CA, USA) with a sensitivity factor of 56.6 × 10^6^ Hz g^−1^ cm^2^. Poly[Ni(CH_3_Salen)] films were deposited on Pt-plated quartz crystals (5 MHz, surface area 1.37 cm^2^). The films were synthesized for several CV cycles at 50 mV s^−1^ between −0.3 V and 0.8 V (vs Ag/AgNO_3_) to obtain charge of synthesis 0.01 C.

The Sauerbrey equation allowed us to calculate the mass change (Δm) during the deposition and testing of the polymer from the shift of resonance frequency (Δ*f*) of the crystal [16] as shown here:

(3) $$\Delta f=\frac{-2f_{0}^{2}}{A\sqrt{p_{q}\mu_{q}}}\Delta m,$$

where *f_0_* is the oscillation frequency of the fundamental mode of the quartz crystal, *A* is the crystal area (1.37 cm^2^), *p_q_* is the density of the quartz (2.648 g cm^−3^) and *μ_q_* is the shear modulus of the quartz (2.947 10^11^ g cm^−1^ s^−2^). These values are specific for the device and in this case can be substituted (4) with the sensitivity factor *C_f_* (56.6 Hz μg^−1^ cm^2^):

(4) $$\Delta f=-C_{f}\Delta m.$$

In the case of charge–discharge of the polymer in the monomer-free solution, the mass changes are caused by counterion injection or repulsion together with the solvent molecules. *M*, g mol^−1^, the apparent molar mass of counterions, can be found from the equation:

(5) $$M=\frac{\partial m}{\partial Q}zF,$$

where *z* is the charge of counterion and *F* is the Faraday constant, 96,500 C∙mol^−1^.

Electrochemical impedance spectroscopy (EIS) and Potentiostatic Intermittent Titration Technique (PITT) experiments were carried out using Metrohm Autolab PGSTAT12 and Metrohm Autolab PGSTAT302N (Eco-Chemie, The Netherlands). For EIS-PITT experiments, nickel–salen-type polymer were deposited onto a glassy carbon (GC) electrode (BASi, geometric area 0.07 cm^2^) by passing 4 mC of polymerization charge through the cell. Details of the experimental data treatment in these methods are presented in the electronic Supplementary Information File.

Conductance of poly[Ni(CH_3_Salen)] film was measured using the Biologic BMP3 potentiostat on Interdigitated Platinum Electrodes (IDE) DRP-G-IDEPT5 (DropSens) (10 mV difference between grids). Cyclic voltammetry at 5 mV s^−1^ was performed simultaneously with conductance measurements. The procedure details are described in [17].

Polymer films for scanning electron microscopy (SEM) measurements were synthesized on the polished glassy carbon plates of various geometric areas by passing 57 mC × cm^−2^ of polymerization charge through the cell. The SEM measurements were performed using a Zeiss Merlin scanning electron microscope at a research park of St. Petersburg State University Interdisciplinary Center for Nanotechnology (Russia).

## 3. Results and Discussion

It has been reported that the charge-transfer process in salen-type polymers at low temperatures is limited by the injection of charge-compensating counterions [18]. The rate of injection depends on the porosity of the film.

Poly[Ni(CH_3_Salen)] films with different porosity were synthesized from electrolyte solutions with different cations of salt. The electrodeposition of the polymers is a ligand-based process in which a radical–radical coupling mechanism is responsible for polymer formation via the linkage of the phenyl rings of neighboring [Ni(CH_3_Salen)] units [19]. The cation radicals participating in the reaction are formed during oxidation of the monomer near the electrode surface. Coupling of cation radicals leads to formation of a solid polymer layer, and this process is accompanied by electrolyte ions becoming trapped inside this layer. Both anions and cations were found to participate in this process, and large cations such as Bu_4_N need larger space between polymer chains or their stacks (Table 1). It was shown that the large diameter of the cation used as a supporting electrolyte in the deposition solution leads to the formation of a large distance between polymer chains (or chain stacks) and thus increases the polymer film porosity [20]. Therefore, the size of micropores in the layers electrodeposited from the [Ni(CH_3_Salen)]-monomer solution increases with the size of the supporting electrolyte cation. Poly[Ni(CH_3_Salen)] films with different porosity were synthesized from electrolyte solutions with different cations of salt.

**Figure 2:**
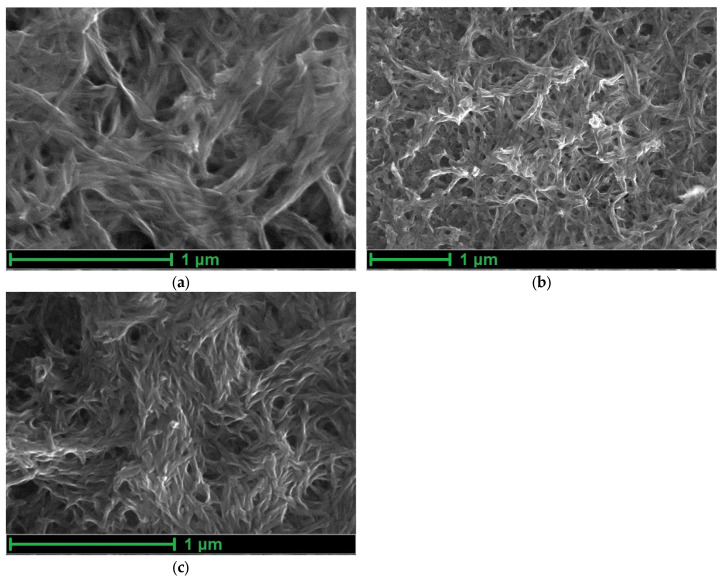
Micrographs of the electrode surface with poly[Ni(CH_3_Salen)] films electropolymerized from monomer solutions with 0.1 M (**a**) Me_4_NBF_4_, (**b**) Bu4NBF4 and (**c**) Bu_4_NTFSI in AN.

Cations of the supporting electrolyte regulate porosity on the micropore level, which cannot be visualized using SEM. However, some effects of the electrolyte composition on the polymer morphology can be visualized (Figure 2). Figure 2a,b show that the film obtained from the Bu_4_NBF_4_ solution is more porous than the film obtained from Me_4_NBF_4_. Here, the anion does not have a noticeable effect on the porosity of the films, and the morphologies of the films polymerized from Bu_4_NBF_4_ and Bu_4_NTFSI are very close (Figure 2b,c).

### 3.1. Charge Transfer Kinetics

Cyclic voltammograms of polymer films of the poly[Ni(CH_3_Salen)] complex were recorded at temperatures of +20 °C, 0 °C, −20 °C and −40 °C at sweep rates from 0.01 to 10 V s^−1^ in different compositions of electrolytes (Me_4_NBF_4_, Bu_4_NBF_4_ and Bu_4_NTFSI in AN). Here, and in the following, films that were synthesized and cycled in the same electrolyte are considered.

**Figure 3:**
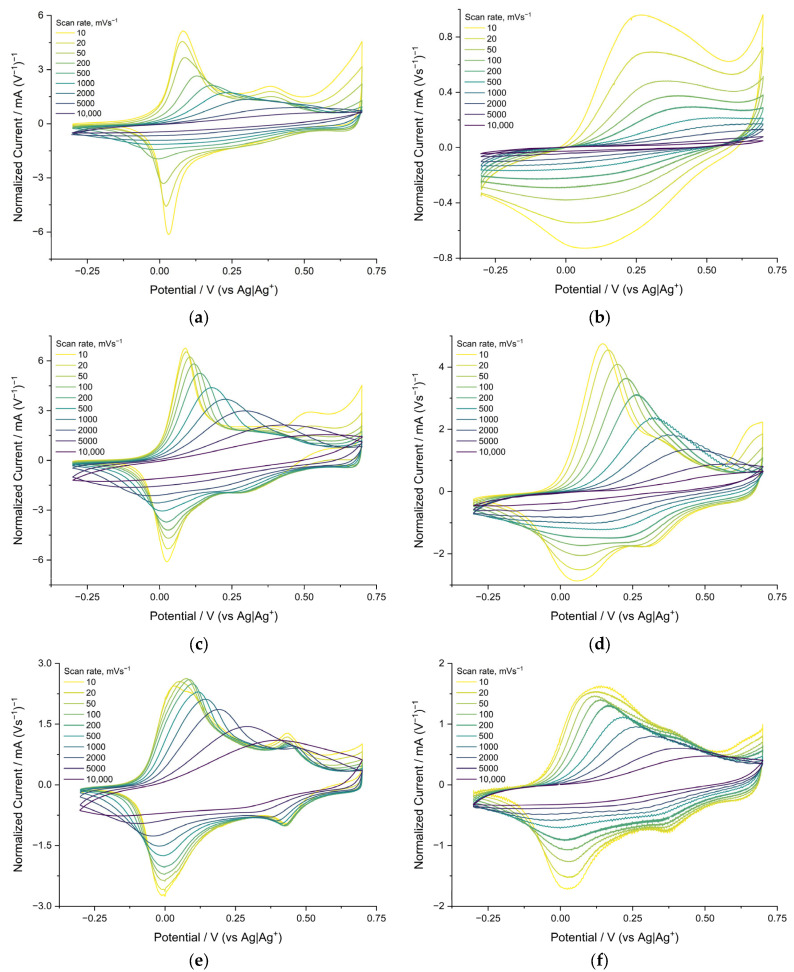
Cyclic voltammograms of poly[Ni(CH_3_Salen)] films synthesized and cycled in 0.1 M Me_4_NBF_4_/AN at (**a**) +20 °C, (**b**) −40 °C; in 0.1 M Bu_4_NBF_4_/AN at (**c**) +20 °C, (**d**) −40 °C; 0.1 M Bu_4_NTFSI/AN at (**e**) +20 °C, (**f**) −40 °C; sweep rates from 0.01 Vs^−1^ to 10 Vs^−1^.

The voltammograms of poly[Ni(CH_3_Salen)] in all electrolytes have typical shapes for this class of materials, as seen from previously published data [18]. The voltammograms of the poly[Ni(CH_3_Salen)] complex recorded in Bu_4_NBF_4_/AN and Me_4_NBF_4_/AN at 20 °C are similar and contain two main pairs of redox peaks around 0 and 0.5 V (Figure 3a,c). The potentials of the anode peaks shift to the right with increasing sweep rate. This shift indicates that the rate of the redox process is limited by the injection of charge carriers into the film. No potential shifts are observed for the reduction peaks, indicating the different nature of the rate-limiting stages of the cathode and anode processes. At a temperature of −40 °C, on the voltammogram of a film cycled in Bu_4_NBF_4_/AN, the main peaks of oxidation and reduction broaden, and the peak currents at a rate of 10 mV/s decrease by 30 and 50%, respectively. The peaks themselves remain on the voltammogram when cycling at speeds below 1 V s^−1^ (Figure 3d). In the case of the Me_4_NBF_4_/AN electrolyte at −40 °C, the voltammogram changes drastically compared to room temperature. The oxidation peaks shift to the right at 0.2 V and disappear at the rates above 1 Vs^−1^. Peak current at 10 mV s^−1^ rate is reduced by 90%. The cathode peak disappears at all sweep rates.

Redox switching in polyNiSalens is associated with oxidative ligand-based processes wherein the positive charge formed during oxidation is delocalized through the polymer chain. There is some evidence of non-direct involvement of the metal in polymer oxidation; however, the polymer is best-described as a polyphenylene-type compound (conducting polymer), rather than an aggregation of nickel complexes (redox polymer), and the main charge carriers are identified as polarons [18,19]. It is known that the oxidation of polyNiSalen-type polymers is accompanied by the injection of anions into the polymer film, which compensate the positive charge formed on the polymer chains, while the reduction is accompanied by the release of anions [12,14]. The features of the voltammograms of the polymer described above suggest that the injection of anions is the slow stage of charge transfer. This injection is either accompanied by anion desolvation or the anion is injected together with the solvation shell. In both cases, this stage can be rate determining, leading to a shift in the anode peaks with an increase in the sweep rate. In contrast, the release of anions into the solution during reduction proceeds rather rapidly, and the potential of the cathodic peaks weakly depends on the scan rate [18].

The voltammograms of the poly[Ni(CH_3_Salen)] complex recorded in 0.1 M Bu_4_NTFSI/AN (Figure 3e) at +20 °C are generally similar to the voltammograms recorded in Bu_4_NBF_4_/AN and Me_4_NBF_4_/AN (Figure 3a,c). They contain two main pairs of redox peaks (about 0 and 0.4 V). However, in this case, the peak currents at the scan rate of 10 mV s^−1^ are 60% lower, and the peaks are twice as wide.

The broadening of CV peaks in the films of conducting polymers indicates an increase in the degree of charge delocalization in the films [21]. Probably such a delocalization occurred because of an increase in the size of the charge-compensating counterion (TFSI^−^ is much larger than BF_4_^−^) and, as a result, a lower polarization of the radical cations by the electric field of the anion that entered the film. The voltammograms of the poly[Ni(CH_3_Salen)] film recorded in Me_4_NBF_4_/AN, Bu_4_NBF_4_/AN and Bu_4_NTFSI/AN at temperatures of 0 °C and −20 °C are presented in the Supporting Information (Figure S1).

The diffusion limitations of charge transfer can be detected from the dependence of the CV peak currents on the sweep rate. In the case of diffusion restrictions, the peak current should be proportional to the square root of the potential sweep rate, and in the absence of them, the peak current should be proportional to the sweep rate. To determine the nature of the dependence, it is convenient to use bilogarithmic coordinates.

**Figure 4:**
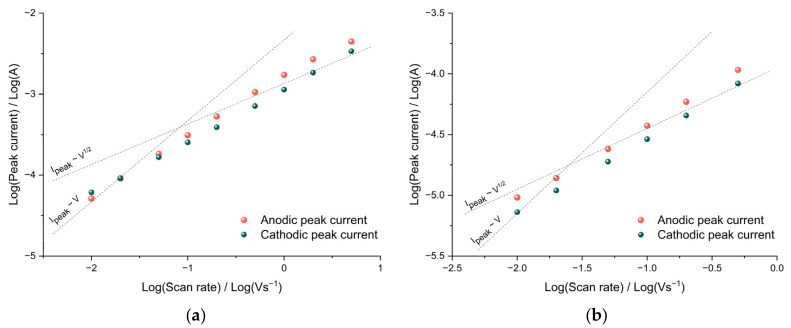

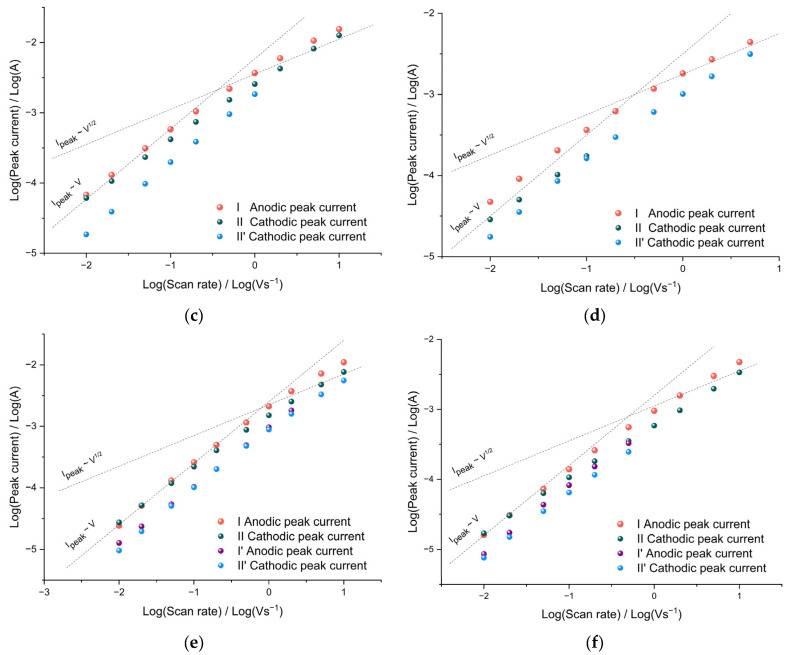
Bilogarithmic dependence of the peak current on the potential scan rate of the [Ni(CH_3_Salen)] film in 0.1 M Me_4_NBF_4_/AN at temperatures (**a**) +20 °C, (**b**) −40 °C, 0.1 M; 0.1 M Bu_4_NBF_4_/AN at temperatures (**c**) +20 °C, (**d**) −40 °C, 0.1 M; Bu_4_NTFSI/AN at temperatures (**e**) +20 °C, (**f**) −40 °C.

As can be seen from the data of poly[Ni(CH_3_Salen)] film cycled in Me_4_NBF_4_/AN, the slope of the bilogarithmic dependences of the oxidation and reduction peak currents on the sweep rate is close to unity at low rates, which indicates the absence of diffusion hindrances in the film both at room temperature and at a temperature of −40 °C (Figure 4a,b). At sweep rates greater than 100 mV s^−1^, the slope decreases to 0.7, indicating the mixed kinetics of the redox process.

It is important to note that an increase in the size of the Bu_4_NTFSI/AN electrolyte salt anion did not lead to the appearance of diffusion restrictions on charge transfer in the film. As can be seen from the bilogarithmic dependences of the peak currents on the sweep rate (Figure 4e,f), at sweep rates from 10 mV s^−1^ to 100 mV s^−1^, the peak current remains proportional to the sweep rate both at room temperature and at −40 °C. Similar to the case of Bu_4_NBF_4_/AN (Figure 4c,d), at sweep rates of more than 200 mVs^−1^, the slope of the dependence decreases to 0.7 over the entire temperature range, indicating a transition to mixed charge transfer kinetics.

The close-to-linear dependence of the peak currents on the sweep rate indicates the occurrence of redox processes by the thin film mechanism; therefore, for all the systems studied, it is possible to calculate the rate constants of the heterogeneous charge-transfer process from the CV data in accordance with Equations (6) and (7) [22]:

(6) $$E_{anodic}{}_{peak}=E^{0^{\prime }}+\frac{RT}{\alpha_{a}zF}\cdot \ln\frac{\alpha_{a}zFv_{a}}{RTk^{0}},$$

(7) $$E_{cathodic}{}_{peak}=E^{0^{\prime }}-\frac{RT}{\alpha_{k}zF}\cdot \ln\frac{\alpha_{k}zFv_{k}}{RTk^{0}},$$

where α is the transfer coefficient; *z* is the number of charges transferred in the anodic or cathodic reaction; *E* and $E^{0^{\prime }}$ are peak and formal potentials, respectively; *R* is the universal gas constant, 8.31 J∙mol^−1^∙K^−1^; *T* is the temperature, K; $\alpha_{a}$, $\alpha_{к}$ are transfer coefficients of the anodic and cathodic reactions; $k_{}^{0}$ is the heterogeneous charge transfer rate constant; *F* is the Faraday constant, 96,500 C∙mol^−1^ and *ν* is the sweep speed, V s^−1^.

The rate constants of the heterogeneous charge-transfer process were calculated for the poly[Ni(CH_3_Salen)] complex during cycling in electrolytes of various compositions.

**Figure 5:**
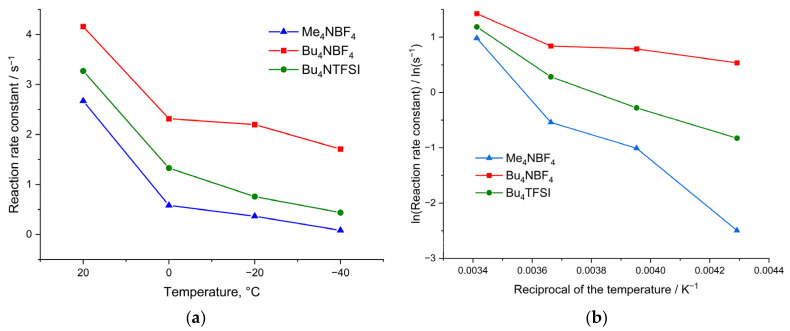
Dependence of the rate constant of the heterogeneous charge transfer in the poly[Ni(CH_3_Salen)] film on temperature upon cycling in Me_4_NBF_4_, Bu_4_NBF_4_ and Bu_4_NTFSI (**a**) in linear coordinates and (**b**) in Arrhenius coordinates.

The values of the calculated rate constant of heterogeneous charge transfer are shown in Figure 5. As can be seen from Figure 5a, the rate constants depend on both the morphology of the polymer film and the size of the counterion, which confirms the suggestion of slow ion injection into the film. Thus, for a film with the smallest pore size (poly[Ni(CH_3_Salen)] synthesized from a monomer solution with the addition of Me_4_NBF_4_ salt), the rate constant is minimal. Furthermore, for the most porous film synthesized from the monomer with the addition of Bu_4_NBF_4_, the rate constants at all temperatures turned out to be maximum.

When cycling in the Bu_4_NTFSI/AN electrolyte, the rate constants are 25% lower than those obtained when cycling in Bu_4_NBF_4_/AN. This clearly indicates that the injection of anion is the limiting stage of the redox process, and replacement of the small BF_4_ by the large TFSI anion leads to a decrease in the injection rate.

However, it is still not clear if the desolvation step is needed to ensure anion injection into the material. To determine the limiting stage of the charge-transfer process at ambient and negative temperatures, we will first discuss the dependence of the rate constants on the reciprocal temperature in the Arrhenius coordinates according to Equation (8):

(8) $$k=Ae^{-E_{a}/(RT)},$$

where *k* is the reaction rate constant, *A* is the Arrhenius constant, i is the activation energy, *R* is the gas constant and *T* is the temperature (K).

The dependences of the rate constant on temperature in Arrhenius coordinates for electrolytes of various compositions are shown in Figure 5b. From Figure 5b, we infer that the dependence for the electrolyte Bu_4_NBF_4_/AN changes with decreasing temperature, and it is impossible to identify unambiguous linear sections on the graph. At temperatures from +20 °C to 0 °C, the slope of the tangent to the rate constant correlates with low activation energy. At more negative temperatures, the slopes of the tangents change, which indicates a change in the activation energy of the injection of counterions into the film. Although these dependences do not contain enough points to obtain accurate numerical values of the activation energy, they can still be used to compare polymer samples with each other at low temperatures. The dependences of the logarithm of rate constant versus reciprocal temperature for films synthesized and measured in the Me_4_NBF_4_ and Bu_4_NTFSI solutions are characterized by only one linear region.

In the case of a film with a sufficient number of large pores, synthesized from Bu_4_NBF_4_/AN, a mechanism with a high activation energy (28 kJ mol^−1^) is realized in the region from +20 °C to 0 °C (Figure 5b). However, as the temperature decreases, a transition to the mechanism with a low activation energy (1.5 kJ mol^−1^ and 3.1 kJ mol^−1^) occurs. This can be explained by the competition between the processes of solvated and desolvated ion injection. At high temperatures, ions are easily desolvated and then they enter into the film trough many micropores. At low temperatures, injection of solvated ions is observed. The possibility of the injection of solvated ions in this case is ensured by the presence of a significant number of large pores in the film of the poly[Ni(CH_3_Salen)] complex synthesized from a monomer solution with the addition of the salt Bu_4_NBF_4_/AN (Figure 2b).

In contrast to polymers with large pores, for a poly[Ni(CH_3_Salen)] film synthesized from a monomer solution with the addition of Me_4_NBF_4_ salt and cycled in the same electrolyte, the dependence of the rate constant on temperature in Arrhenius coordinates has only one slope and a high activation energy of 26 kJ mol^−1^, which indicates the dominance of the injection process with desolvation in a wide temperature range. This is probably due to the absence of a significant number of large pores and the impossibility of injection of the solvated ion (Figure 2a). A similar type of dependence of the rate constant on temperature in Arrhenius coordinates can be observed for the poly[Ni(CH_3_Salen)] film using the Bu_4_NTFSI/AN deposition and testing electrolyte. In this case, the dependence has only one slope and the activation energy is 18 kJ mol^−1^. Although the poly[Ni(CH_3_Salen)] film synthesized using this electrolyte is quite porous, the use of the TFSI^−^ counterion has a larger radius compared to BF_4_^−^ and cannot enter any pore in a solvated state. This leads to the implementation of the charge transfer mechanism with desolvation. Thus, the CV analysis at different scan rates showed that the injection of counterion into the poly[Ni(CH_3_Salen)] film limits the charge-transfer process. The mechanism of injection and the activation energy of this process depend on the temperature, properties of the film and on the composition of the electrolyte. Polymers with high porosity work most efficiently in electrolytes with small counterions.

### 3.2. Mass Transfer

To confirm the limitation of the charge-transfer process by the counterion injection stage, the mass transfer parameters were determined in a wide temperature range (from +20 °C to −40 °C) for a poly[Ni(CH_3_Salen)] film deposited using Me_4_NBF_4_/AN, Bu_4_NBF_4_/AN and Bu_4_NTFSI/AN. The polymer film masses, m_QCM_, were calculated using the QCM experimental data using the Sauerbrey Equation (3) 3.62 µg, 4.28 µg and 3.26 µg for Me_4_NBF_4_/AN, Bu_4_NBF_4_/AN and Bu_4_NTFSI/AN, respectively. The thicknesses of the obtained polymers were approximately calculated according to the procedure described in [23] as 1.39 µm, 1.64 µm and 1.39 µm for Me_4_NBF_4_/AN, Bu_4_NBF_4_/AN and Bu_4_NTFSI/AN, respectively.

By means of the method of electrochemical quartz crystal microbalance (EQCM), the mass of charge-compensating electrolyte anions with their solvate shells was determined. The apparent mass of the charge-compensating ion will make it possible to assess its solvation, which allow us to confirm the assumptions formulated in the previous section on the nature of counterion injection using the results of direct measurements.

**Figure 6:**
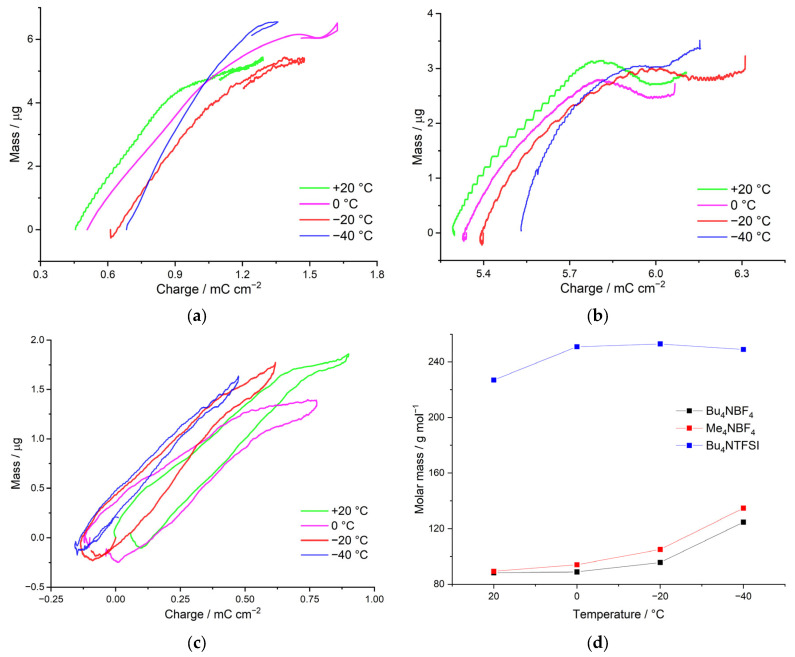
Massograms of the poly[Ni(CH_3_Salen)]-modified electrode, deposition and cycling electrolyte (**a**) Me_4_NBF_4_/AN, (**b**) Bu_4_NBF_4_/AN, (**c**) Bu_4_NTFSI/AN with a temperature range from +20 °C to −40 °C and (**d**) with temperature dependence of the molecular weight of the charge-compensating counterion.

We consider the dependence of the molecular weight of the charge-compensating ion on the temperature for poly[Ni(CH_3_Salen)] complex in the deposition and cycling electrolytes with Me_4_NBF_4_/AN, Bu_4_NBF_4_/AN and Bu_4_NTFSI/AN salts. As can be seen from the dependences presented, in the case of the Me_4_NBF_4_/AN-based electrolyte, the counterion mass of the counterion is 110 g mol^−1^, which is close to the mass of the intercalating BF_4_^–^ anion (87 g mol^–1^) with one AN molecule (41 g mol^–1^). The mass of the counterion in this case practically does not change with decreasing temperature. When Bu_4_NTFSI/AN is used as an electrolyte, the counterion mass also does not depend on temperature, and in this case is 227 g mol^−1^, which probably corresponds to the injection of the TFSI^−^ anion (280 g mol^−1^) and the repulsion of the AN molecule (Figure 6a). Thus, in both cases, the transfer of unsolvated ions occurs.

In the case of using the Bu_4_NBF_4_/AN electrolyte in synthesis, the dependence of the counterion molecular weight on the temperature has two linear segments. In the area from +20 °C to 0 °C, the mass of the injecting ion is 325 g mol^−1^, which probably corresponds to the injection of the BF_4_^−^ anion (87 g mol^−1^) and 5 AN molecules (41 g mol^−1^). With a further decrease in the temperature, the mass of the counterion increases sharply to 706 g mol^−1^ at −40 °C, which corresponds to the injection of an even-more solvated anion (Figure 6b).

Thus, the change in the type of mass transfer at low temperatures is consistent with the change in the rate constant. For example, for the electrolytes Me_4_NBF_4_/AN and Bu_4_NTFSI/AN, injection of desolvated BF_4_^−^ and TFSI^–^ counterions, respectively, is observed throughout the temperature range. Desolvation of ions in this case is confirmed not only by the low mass, but also by the high activation energy. In the case of Bu_4_NBF_4_/AN, the Arrhenius dependence of the rate constant on temperature exhibits two linear segments. At temperatures of +20 °C to 0 °C, the nonsolvated anion is injected, which is a high activation energy process. At temperatures from 0 °C to −40 °C, the mass of the counterion increases sharply and the activation energy decreases, indicating the injection of a solvated ion.

### 3.3. Electronic Conductance

**Figure 7:**
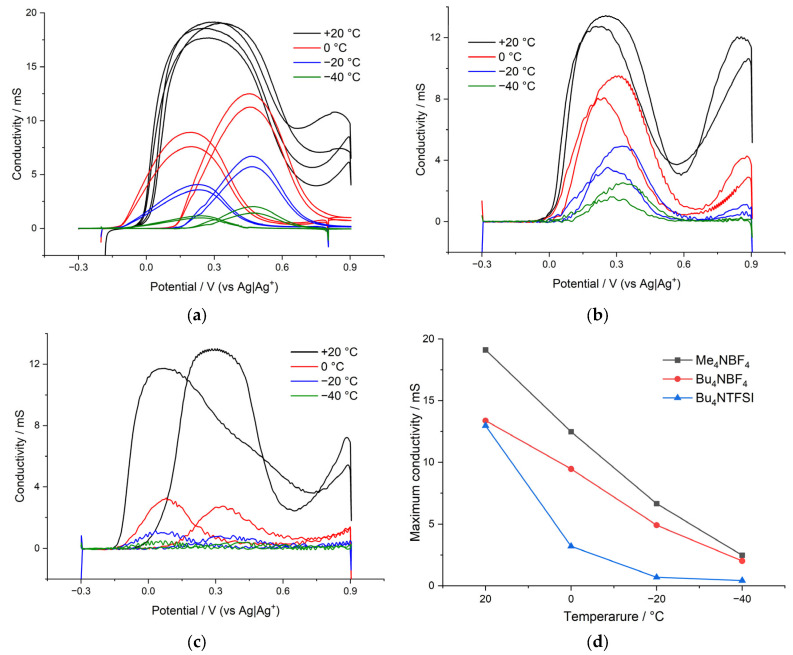
Electronic conductance of the poly[Ni(CH_3_Salen)] complex at temperatures from 0 °C to −40 °C recorded in (**a**) Me_4_NBF_4_/AN, (**b**) Bu_4_NBF_4_/AN and (**c**) Bu_4_NTFSI/AN. (**d**) Temperature dependence of the maximum conductance.

The electronic conductance of the poly[Ni(CH_3_Salen)] complex was studied in the temperature range from +20 °C to −40 °C on interdigitated electrodes. Me_4_NBF_4_, Bu_4_NBF_4_ and Bu_4_NTFSI were used as the salts in the deposition and cycling electrolytes. (Figure 7).

Figure 7 shows the temperature dependence of the maximum conductance in various electrolytes. As can be seen from the figure, the poly[Ni(CH_3_Salen)] film synthesized from a monomer solution with the addition of Me_4_NBF_4_ is characterized by the densest packing, which leads to an increase in its conductance compared to a more porous film synthesized from Bu_4_NBF_4_ (18 and 13 mS, respectively) (Figure 7d). At room temperature, the replacement of the electrolyte salt with Bu_4_NTFSI has practically no effect on the value of the maximum conductance. However, as the temperature decreases, the conductance of this film decreases sharply from 13 mS to 2 mS already at 0 °C. Thus, the conductance of the poly[Ni(CH_3_Salen)] complex, both at room and at negative temperatures, is affected by both the synthesis electrolyte and the cycling electrolyte; polymer films with a denser packing of layers are characterized by the maximum conductance (Figure 7d).

### 3.4. Impedance Spectroscopy and Potentiostatic Intermittent Titration

Analysis of electrochemical responses of poly[Ni(CH_3_Salen)] electroactive polymer film in different 0.1 M electrolytes (Bu_4_NTFSI, Me_4_NBF_4_, Bu_4_NBF_4_ in acetonitrile solution) at different temperatures (+20 °C, 0 °C, −20 °C и −40 °C) were conducted by means of electrochemical impedance spectroscopy (EIS) and the potentiostatic intermittent titration technique (PITT).

The difficulty of estimating diffusion coefficients for the porous polymer materials with several charge carriers arises from the lack of knowledge on the double-layer structures, pore size distributions, pore effective volume, tortuosity, potential distribution within the polymer, electrolyte distribution within the film domains, etc. However, despite all of this, the widespread approach of using the Cottrell equation to estimate the apparent diffusion coefficients of the polymer film can still be applied. In this case, the film is treated as a single-phase system and the result of such an approach would be the apparent diffusion coefficients, which include implicitly all the above-mentioned factors complicating the diffusion. The apparent diffusion coefficients were calculated from PITT data in the region between –0.2 and 0.6 V vs. Ag|Ag^+^, considering the finite nature of the diffusion problem in a polymer film with an estimated thickness of 800 nm.

**Figure 8:**
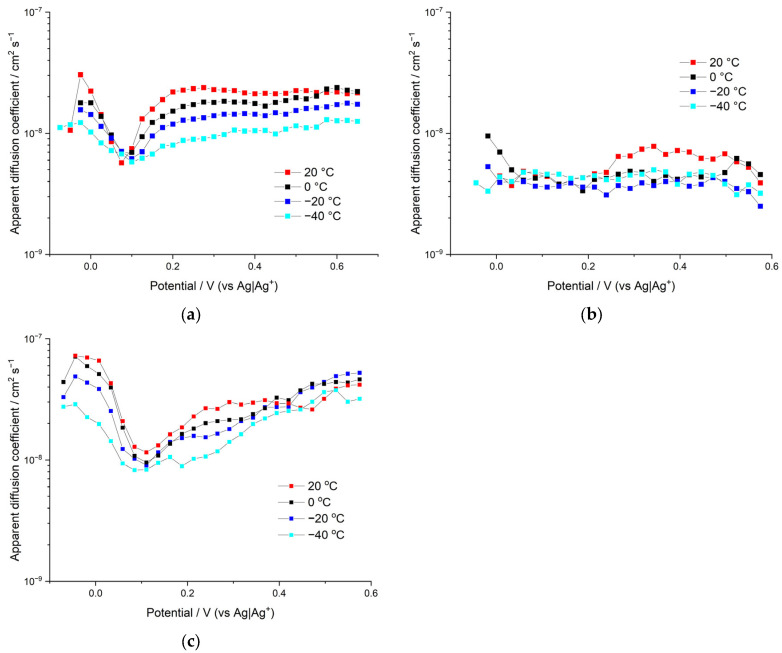
Apparent diffusion coefficients of polymer film poly[Ni(CH_3_Salen)] in different 0.1 M electrolytes (**a**) Me_4_NBF_4_; (**b**) Bu_4_NTFSI; (**c**) Bu_4_NBF_4_ in acetonitrile solution at different temperatures (+20 °C, 0 °C, −20 °C and −40 °C).

Apparent diffusion coefficients *D_App_* of poly[Ni(CH_3_Salen)] electroactive polymer films synthesized and tested in different 0.1 M electrolyte solutions are demonstrated in Figure 8.

The apparent diffusion coefficients for the sample synthesized and measured in Bu_4_NBF_4_ are the highest of all the samples, reaching relatively high values of 7 × 10^−8^ cm^2^/s at the fully discharged state of the films (in the beginning of film electroactivity) and 5 × 10^−8^ cm^2^/s at the charged state of the films. The dependence of *D_App_* on the applied potential is not linear; a sharp minimum of diffusion coefficients was observed in the proximity of the formal potential of the polymer redox peak. This is a characteristic feature of many redox-active materials, and it can be understood in terms of the attractive interactions within the film and the corresponding change in the activity coefficients of charge-transfer species [24]. Influence of the temperature on the apparent diffusion coefficient is rather moderate. Therefore, a significant decrease in temperature (from 20 °C to −40 °C) results in a ~2–3 times decrease in the measured coefficients.

Similar behavior of apparent diffusion coefficients *D_App_* on the temperature and the applied potential is observed for the sample synthesized and measured in Me_4_NBF_4_ solution (see Figure 8). Thus, this sample reaches its highest *D_App_* value at a fully discharged state at negative potentials (*D_App_* = 3 × 10^−8^ cm^2^/s) and at a charged state at positive potentials *D_App_* = 2.5 × 10^−8^ cm^2^/s. Additionally, similar to the previously described sample, the minimum on the *D_App_* vs. potential dependence occurs at the vicinity of the formal potential, wherein the *D_App_* is down to around 6 × 10^−9^ cm^2^/s. Thus, both polymer films in Bu_4_NBF_4_ and Me_4_NBF_4_ electrolytes demonstrate a sharp and profound minimum (approximately one order of magnitude) in the proximity of redox peak potential of their CV curves. Their CV curves demonstrate sharp redox-peaks (at slow scan rates mid-width ~100 mV) that can be attributed to the strong attraction of redox sites within the film [25,26], which leads to the minimum in activity coefficients, which in turn leads to the minimum in the measured diffusion coefficients.

The overall conclusion of the above comparison is that the sample with larger pores (deposited in Bu_4_NBF_4_ solution) demonstrates better diffusion characteristics, provided that the counterion size is the same.

In contrast to both samples described above, CV curves of polymer film electrodeposited and tested in Bu_4_NTFSI electrolyte solution demonstrate wider voltammetric peaks (~200 mV) that can be attributed to less attractive interaction within the film and correspondingly fewer changes in activity coefficients with the potential. Insignificant change in activity coefficients provides smooth and hardly distinguishable minimum of diffusion coefficients around the redox peak potential of this sample. Thus, the apparent diffusion coefficient for the sample synthesized and measured in Bu_4_NTFSI starts from relatively high values of around 1 × 10^−8^ cm^2^/s at a fully discharged state of the film (in the beginning of the film electroactivity), then undergoes a smooth minimum of ~4.5 × 10^−9^ cm^2^/s (between 0.05 and 0.22 V) and then grows to around 6 × 10^−9^ cm^2^/s. Similar to both previous samples, the *D_App_* of this sample shows insignificant dependence on the temperature (*D_App_* decreases ~2–3 times per 60 °C temperature decrease). The overall conclusion from the above is that larger anion size deteriorates the diffusion characteristics of the sample, provided that porosity remains the same.

A detailed description of the diffusion coefficient determination is provided in the Supplementary Information (Figures S2 and S3).

Next, we analyze the differences in the charge-transfer resistances for poly[Ni(CH_3_Salen)] polymer films electrodeposited and tested in different electrolytes, as kinetic polarization can result in significant potential shift during CV cycling and deterioration of the overall electroactive film performance. Charge-transfer resistances R_CT_ and electric double-layer capacities C_EDL_ of the above-mentioned electrochemical systems were estimated by means of electrochemical impedance spectroscopy (EIS).

**Figure 9:**
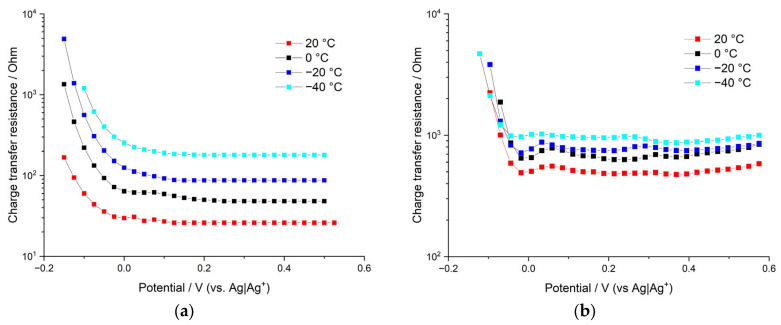
Charge-transfer resistances of polymer film poly[Ni(CH_3_Salen)] in different 0.1 M electrolytes in acetonitrile solution (**a**) Me_4_NBF_4_; (**b**) Bu_4_NBF_4_. Different temperatures (+20 °C, 0 °C, −20 °C and −40 °C).

We should underline here that the charge-transfer resistances extracted from EIS measurements correspond to so-called low-overpotential resistances, as they are measured with low-amplitude sinusoidal potential perturbations (amplitude is 5 mV) imposed on a steady value of the potential. At high overpotentials (which take place, for example, at CV measurements at high potential scan rates) charge-transfer resistances are different. Nevertheless, EIS can still be used as a tool to check the temperature dependence of low-overpotential *R_CT_* and to estimate the values of electric double-layer capacity of the film. Besides, if we assume that the prevailing mechanism of ion intercalation (solvated ion intercalation or desolvation followed by intercalation of desolvated ion) is independent of the overpotential (while the rate of the ion intercalation is, of course, dependent on the overpotential), we can expect that temperature dependence of 1/*R_CT_* measured by EIS technique should, in principle, coincide with temperature dependence of heterogeneous rate constants measured using the CV method. Figure 9 demonstrates *R_CT_* vs. electrode potential dependence for poly[Ni(CH_3_Salen)] film in 0.1 M Bu_4_NBF_4_/AN and Me_4_NBF_4_/AN. It is clearly seen that both systems demonstrate a sharp decrease in charge-transfer resistances: R_CT_ starts from very high values at nonelectroactive potentials (where the conductance is very low), goes down to the minimum value at the vicinity of redox peak potential, and then stays nearly constant around this value and up to the high positive potentials. Thus, the asymmetry (relative to the formal potential of the main redox peak) in the behavior of *R_CT_* is observed for both samples. This sheds light on the asymmetric behavior of anodic and cathodic CV peaks measured at high potential scan rates. Anodic CV branches of all samples start at negative potentials (which correspond to high values of *R_CT_*). Thus, high starting values of *R_CT_* to the left of the main redox peak correlate with the significant shift of the anodic peak potential at high potential scan rates. On the other hand, cathodic CV branches of all samples start at positive potentials, which correspond to low values of *R_CT_*. This correlates with insignificant shifts of cathodic CV-peaks at high potential scan rates. The asymmetry of the *R_CT_* values relative to the main redox peak potential can be understood in the following way. In the discharged state (to the left of the main peak) film is relatively compact and dense as it does not a have sufficient number of intercalated anions yet. However, an increase in the applied potential leads to the beginning of intercalation, which, in turn, leads to swelling of the film and to opening of pores and crevices of the polymer domains, resulting in low *R_CT_* values.

The lowest charge-transfer values are observed for the film deposited and measured in Me_4_NBF_4_/AN solution at room temperature. However, *R_CT_* of this sample grows quickly with the decrease in temperature (10-fold increase in *R_CT_* per 60 °C decrease in temperature). Such strong temperature dependence corresponds to around 23 kJ/mol activation energy, which is in agreement with the activation energy of ion intercalation calculated for this sample from the shift of CV-peak potentials at different potential scan rates (26 kJ/mol). The other sample (deposited and measured in the Bu_4_NBF_4_/AN solution) shows higher values of charge-transfer resistance at positive potentials. However, the growth of *R_CT_* values with decreasing temperature is much slower in this case; only a two-fold increase in *R_CT_* is observed for 60 °C temperature decrease. This temperature dependence corresponds to approximately 5.5 kJ/mol activation energy, which is in agreement with the average value obtained from the measurements of the shift of CV-peak potentials (2.5 times decrease in the heterogeneous rate constant per 60 °C corresponds to the activation energy of 8.8 kJ/mol). Thus, estimations of activation energies of ion intercalation based on both CV and EIS measurements give consistent results. This is in good agreement with the main assumption of this work, that is, ion intercalation limits the charge-transfer kinetic. Anion intercalation is less energy-costly for the film deposited in Bu_4_NBF_4_/AN solution, as this sample has the largest pores. Therefore, the activation energy of intercalation is the smallest one for this sample, which, in turn, results in less temperature-sensitive charge-transfer resistance in this sample. The opposite case is the film deposited in Me_4_NBF_4_/AN solution; this sample has the smallest pores, which results in higher activation energy of intercalation and sharper dependence of the charge-transfer resistance on the temperature.

**Figure 10:**
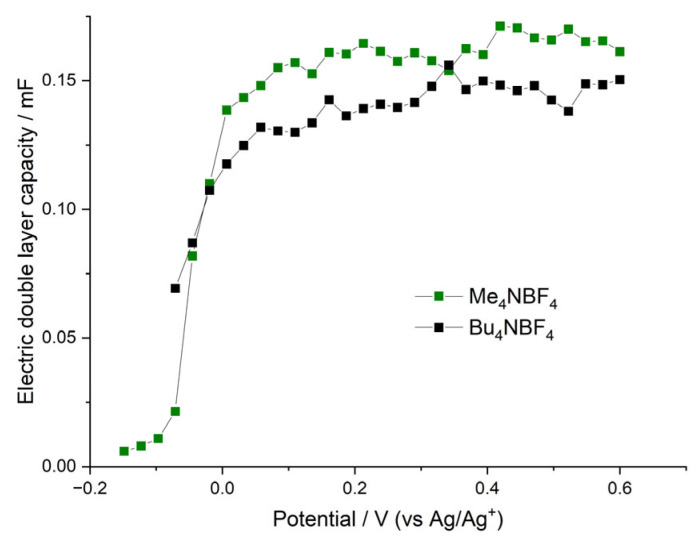
Dependence of electric double-layer capacities of the polymer film poly[Ni(CH_3_Salen)] on the applied potential. The green line represents the film electrodeposited and tested in 0.1 M Me_4_NBF_4_/AN solution. The black line represents the film electrodeposited and tested in 0.1 M Bu_4_NBF_4_/AN solution.

The only system that has a significant amount of pores with sizes larger than the size of solvated anions is the film deposited and tested in Bu_4_NBF_4_/AN solution. During the electrodeposition process, Bu_4_N^+^ cations form a sufficient number of pores of a high radius, which allow small BF_4_^−^ anion penetration with partial desolvation or without desolvation at all. Therefore, a decrease in temperature does not cause a significant energy cost for anion uptake in the case of the film electrodeposited in Bu_4_NBF_4_/AN solution, as long as the intercalation mechanism without desolvation becomes more beneficial. The film deposited and tested in the Me_4_NBF_4_/AN solution is capable of uptaking only desolvated BF_4_^−^ anions (as solvated BF_4_^−^ anions do not fit the pores formed by small Me_4_N ^+^ ions during electrodeposition); this results in continuous growth of the energy cost of anion intercalation with decreasing temperature and continuous growth of R_CT_ values. Thus, estimations of the change in the reaction rate constants with temperature (Figure 5) are in quantitative agreement with the estimation of *R_CT_* changes (Figure 9). Thus, the conclusion that desolvation limits the charge-transfer kinetic at low temperatures for samples with small pores is confirmed again. However, for samples with bigger pores (tested in Bu_4_NBF_4_/AN), desolvation has only limited influence over charge-transfer kinetic. Electric double-layer capacities of the above systems demonstrate sharp growth with increasing electrode potential (in the vicinity of the redox peak potential), which is illustrated in Figure 10. This growth coincides with the beginning of the electroactivity of the film, which, in turn, coincides with the sharp decrease in the charge-transfer resistance. At potentials higher than the redox peak potential, *C_EDL_* reaches its maximum value. This behavior can be understood in the following way. The first redox CV peak, as we mentioned above, corresponds to the oxidation of the fragments of the polymer with simultaneous intercalation of the charge compensating anions into the film. However, the anion intercalation leads to overall film swelling and opening of the film pores and crevices, which, in turn, leads to the sharp increase in the film double-layer capacity and to a decrease in the film charge-transfer resistance. That is why the polymer film has its own non-faradaic part of capacity, which becomes active together with the beginning of the film electroactivity around the redox peak potential. The maximum *C_EDL_* values of both samples are comparable and reach around 0.15 mF (a slightly higher value is observed for the film deposited and tested in the Me_4_NBF_4_/AN solution). Observed values of electric double-layer capacities are rather high and imply that a significant part of the film-charging current at potentials higher than the redox peak potential corresponds to the so-called nonfaradaic current of the double-layer charging. A detailed description of the double-layer capacity value determination and charge-transfer resistance determination is provided in the Supplementary Information (Figures S4–S7).

## 4. Conclusions

We investigated the kinetic, mass and charge transfers in the polymer film of poly[Ni(CH_3_Salen)] synthesized in Me_4_NBF_4_, Bu_4_NBF_4_ and Bu_4_NTFSI. Temperature-dependent relationships of poly[Ni(CH_3_Salen)] charge-transfer parameters in acetonitrile-based electrolyte solutions with Me_4_NBF_4_, Bu_4_NBF_4_ and Bu_4_NTFSI were determined. It was shown that injection of counterions into polymer film limits the redox processes, and the activation energy of the process depends on the film structure as well as electrolyte composition. Investigation of the mass and charge-transfer parameters under the same circumstances proved the limitation. On the basis of the temperature dependence of charge-transfer characteristics, we concluded that there are two types of counterion injection processes that take place during polymer electrooxidation: one is the injection of solvated anions, and the other is the desolvation step on the polymer layer surface followed by the injection of desolvated anions. The first process has low activation energy, but it also has a low Arrhenius constant, and so at positive temperatures, the second mechanism is preferable. At subzero temperatures, injection of solvated ions dominates during the ionic charge transfer. However, this leads to a low rate of charge transfer due to kinetic limitations. The predominant injection mechanism depends both on the properties of the film and the composition of the electrolyte. Polymers with high porosity demonstrate fast ionic charge transfer, being able to inject solvated ions even at temperatures as low as −40°C. Polymers with low porosity demonstrate significant deterioration of their charge-transfer characteristics at subzero temperatures. This finding may be used to optimize the composition of energy storage devices, such as supercapacitors and batteries based on polymeric charge-storage materials.

## Figures and Tables

**Table 1:** Diameters of cations of supporting electrolytes.

Cation Type	Diameter, Å
(Bu_4_N)^+^	9.6
(Me_4_N)^+^	4.1

## Data Availability

Data available from the corresponding authors from request.

## Supplementary Materials

The following supporting information can be downloaded at: https://www.mdpi.com/article/10.3390/polym15051323/s1, Figure S1: Cyclic voltammograms of poly[Ni(CH_3_Salen)] films synthesized and cycled in 0.1M Me_4_NBF_4_/AN (a) 0 °C, (b) −20 °C; in 0.1 M Bu_4_NBF_4_/AN at (c) 0 °C, (d) −20 °C; 0.1 M Bu_4_NTFSI/AN at (e) 0 °C, (f) −20 °C; sweep rates from 0.01 V s^−1^ to 10 V s^−1^; Figure S2: *I* vs. *t* of poly[Ni(CH_3_Salen)] synthesized and measured in 0.1 M Bu_4_NTFSi/AN electrolyte solution; Figure S3: I_*_t^1/2^ vs. t of poly[Ni(CH_3_Salen)] synthesized and measured in 0.1 M Bu_4_NTFSi/AN electrolyte solution; Figure S4: Nyquist plot of the poly[Ni(CH_3_Salen)] film synthesized in 0.1 M Me_4_NBF_4_/AN solution and recorded in 0.1 M Me_4_NBF_4_/AN solution at 0 °C at 0.190 V (vs. Ag|Ag^+^); Figure S5: Equivalent scheme used for R_CT_ and C_EDL_ determination; Figure S6: Examples of impedance spectra of poly[Ni(CH_3_Salen)] film recorded in Bu_4_NBF_4_/AN solution at different temperatures at 0.200 V (vs. Ag|Ag^+^); Figure S7: Examples of impedance spectra of poly[Ni(CH_3_Salen)] film recorded in Me4NBF4 /AN solution at different temperatures at 0.190 V (vs. Ag|Ag^+^). References [27,28,29,30,31] are cited in the Supplementary Materials.
