# Supplementary material for: Mass and Charge Transfer in a Polymeric NiSalen Complex at Subzero Temperatures
Source: Polymers (Basel). 2023 Mar 6;15(5):1323. doi: 10.3390/polym15051323 (PMC10007232; doi:10.3390/polym15051323)
Supplement: Supplementary file 1 [file polymers-15-01323-s001.zip › polymers-2168136-supplementary.pdf]

Article

Mass and charge transfer in a polymeric NiSalen complex at subzero temperatures

Elena V. Alekseeva*, Julia V. Novoselova, Dmitrii V. Anischenko, Vasiliy V. Potapenkov, and Oleg V. Levin*

[1] Institute of Chemistry, Saint Petersburg University, 7/9 Universitetskaya nab., St. Petersburg 199034, Russia

* **Correspondence: E.A.** e.v.alekseeva@spbu.ru; Tel.:+7-921-655-74-97; O. L. o.levin@spbu.ru

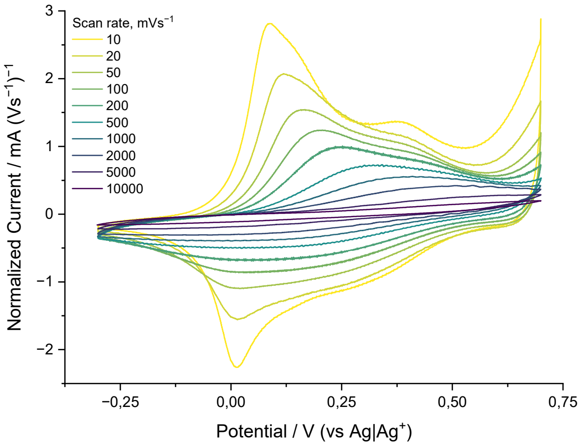

(**a**)

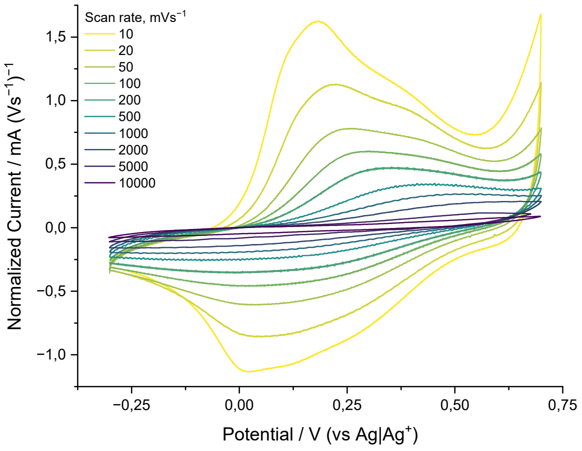

(**b**)

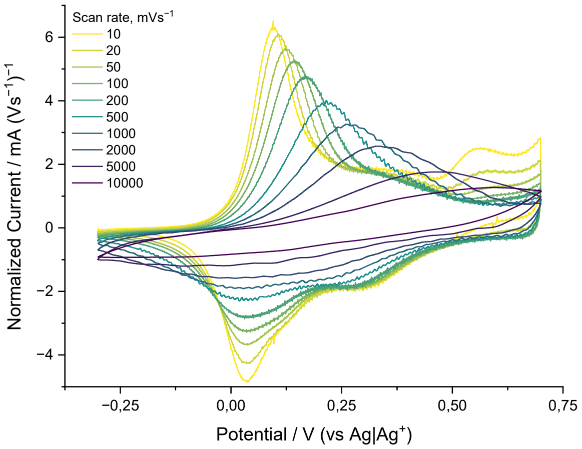

(**c**)

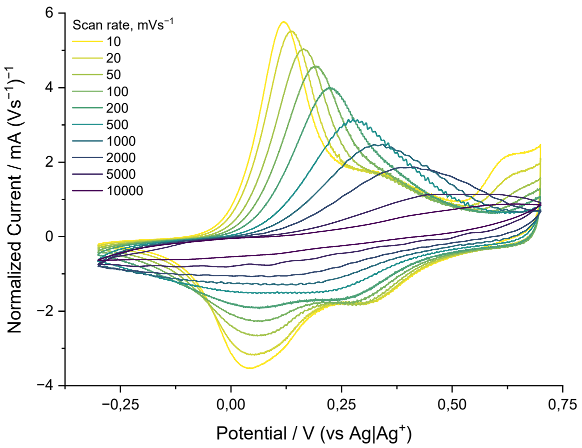

(**d**)

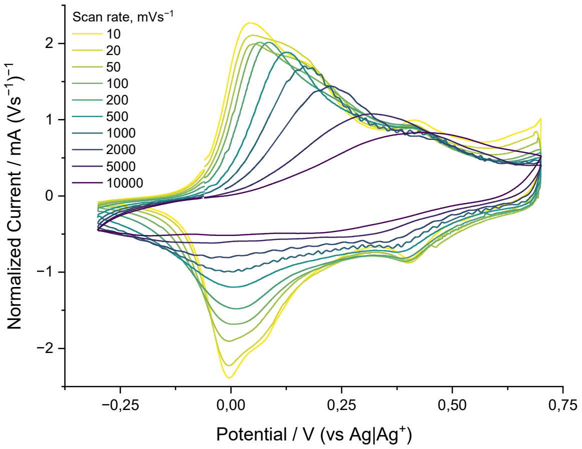

(**e**)

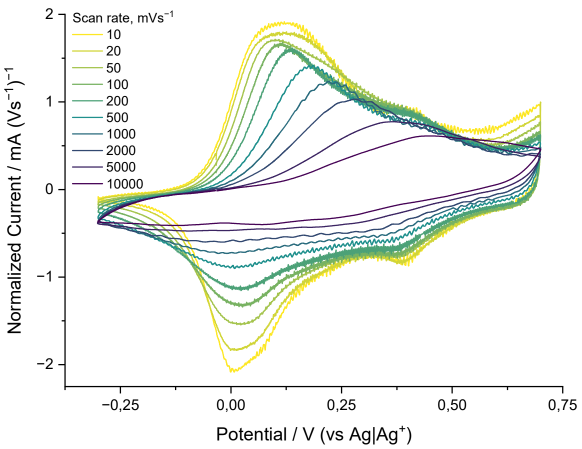

(**f**)

Fig. S1. Cyclic voltammograms of poly[Ni(CH_3Salen)] films synthesized and cycled in 0.1M Me_4NBF_4/AN (**a**) 0°C, (**b**) −20°C; in 0.1M Bu_4NBF_4/AN at (**c**) 0°C, (**d**) −20°C; 0.1M Bu_4NTFSI/AN at (**e**) 0°C, (**f**) −20°C; sweep rates from $0.01Vs^{-1}$ to $10Vs^{-1}$.

We used widespread approach for determination of the apparent diffusion coefficients for materials undergoing ion intercalation [1s-3s]. Diffusion coefficients of all samples and their dependence on the electrode potential were determined from PITT measurements using the Cottrell equation (eq.1s.)

$$D_{App} = \frac{(I\sqrt{t})_{MAX}{}^{2} L^{2} \pi}{\Delta Q^{2}} \qquad 1s$$

Where ΔQ is the charge caused by a potential step, D_{App} is the apparent diffusion coefficient, $(I\sqrt{t})_{MAX}$ is the maximum value of the plot ($I{*}t^{1/2}$ vs. t), L is the film thickness, π is 3.14.

Here we provide an example of a current transient (I vs. t) of poly[Ni(CH_3Salen)] synthesized and measured in 0.1M Bu_4NTFSi/AN electrolyte solution at 0^0C. Transient recorded at 0.290 V vs. Ag|Ag^+.

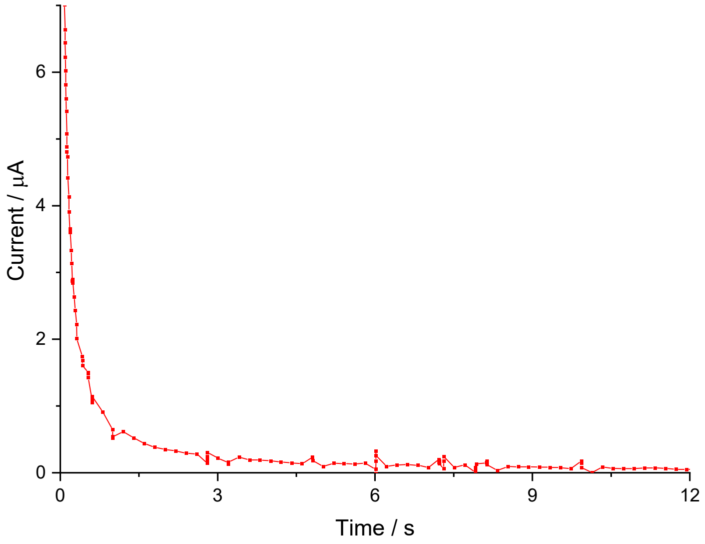

Fig.S2. I vs. t of poly[Ni(CH_3Salen)] synthesized and measured in 0.1M Bu_4NTFSi/AN electrolyte solution. Transient recorded at 0^0C, 0.290 V vs. Ag|Ag^+. Thus, by integration of the transient one gets the ΔQ value, which can further be substituted in eq.1s. For given step ΔQ = 0.0043mC.

In order to find $(I\sqrt{t})_{MAX}$, one should plot $I{*}t^{1/2}$ vs. t dependence (Fig.S3).

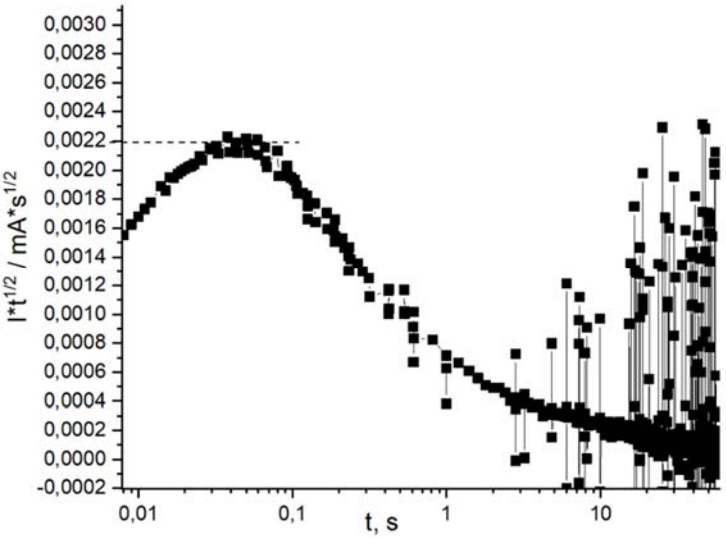

Fig.S3. $I{*}t^{1/2}$ vs. t of poly[Ni(CH$_3$Salen)] synthesized and measured in 0.1M Bu$_4$NTFSi/AN electrolyte solution. Transient recorded at 0^0C, 0.290 V vs. Ag|Ag$^+$. For given step $(I\sqrt{t})_{MAX}$ = 0,0022 [mA$*$s$^{1/2}$].

Estimations of the film thickness gives *L* based on the charge spent on the synthesis gives ~800 nm. Therefore, substituting $(I\sqrt{t})_{MAX}$, ΔQ and *L* values into the eq.1s, one gets the value of the apparent diffusion coefficient D_{App} = $5{*}10^{-9}$ cm^2/s.

EIS was used for charge transfer resistance and double layer capacitance determination (Fig.S4). One may notice that impedance spectra of polymers have inclined straight-line instead of pure capacitance π/2 vertical line at low frequencies. Additionally, spectra do not contain π/4 typical Warburg line. Hence, extracting diffusion characteristic by means of EIS are complicated. Use of anomalous diffusion or modified restricted diffusion can, in theory, help extracting diffusion characteristics from EIS [4s-6s]. However, interpretation and reliability of these approaches are still questionable as they provide different results and have multiple possible good fits for almost each spectrum. That is why we decided to use relatively simple equivalent scheme (Fig.S5) to avoid interpretational issues and reliability issues. However, the immediate drawback of the scheme Fig.S5 is that it approximates experimental data with a good quality only at high and medium frequency regions (Fig.S4, at low frequencies the discrepancy between the data and the fit is growing). However, one can still focuses on high and medium frequencies of impedance spectra in order to determine R_{CT} and C_{EDL} of the polymer film. We used the following equivalent scheme for R_{CT} and C_{EDL} determination (Fig.S5).

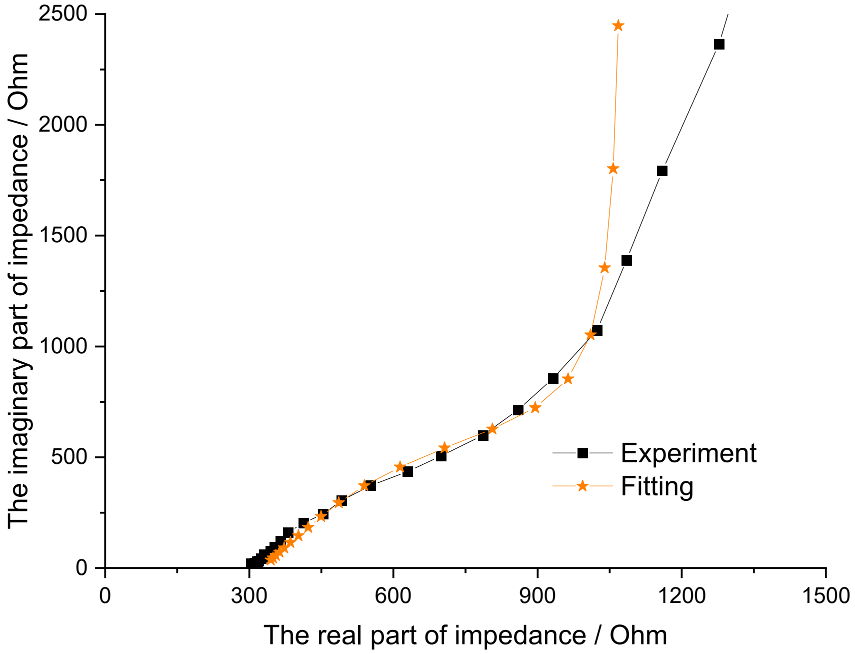

Fig.S4. Nyquist plot of the poly[Ni(CH$_3$Salen)] film synthesized in 0.1M Me$_4$NBF$_4$/AN solution. Recorded in 0.1M Me$_4$NBF$_4$/AN solution at 0°C at 0,190 V vs. Ag|Ag$^+$. The fitted values of parameters are following: charge transfer resistance R_{CT} =673 Ohm, electric double layer capacitance C_{EDL} = 108mF, solution resistance R_{SOL} = 320 Ohm, characteristic diffusion time t_d = 0,067 s, diffusion resistance R_d = 271 Ohm. Electrode potential 0.190V.

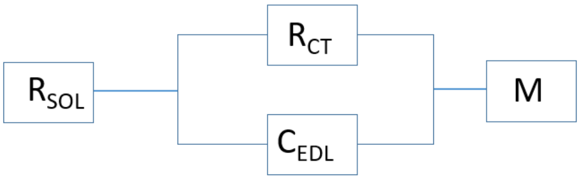

Fig.S5. Equivalent scheme used for R_{CT} and C_{EDL} determination. R_{SOL} is the resistance of the electrolyte solution; M is the restricted diffusion element, which accounts for the fact that diffusion occurs within the finite film media; this element includes two parameters: diffusion resistance R_d and characteristic diffusion time t_d.

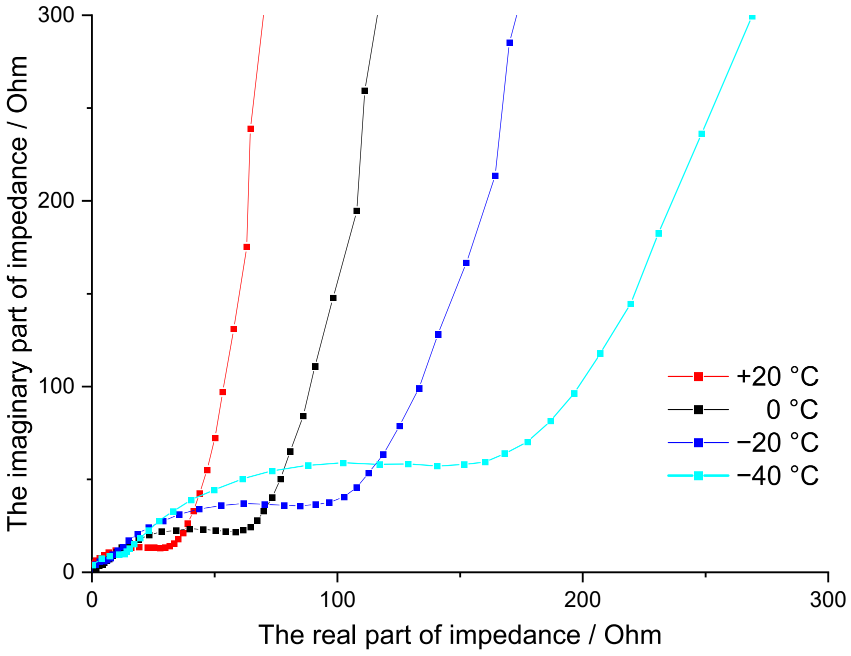

Fig.S6. Examples of impedance spectra of poly[Ni(CH_3Salen)] film recorded in Bu_4NBF_4/AN solution at different temperatures at 0.200 V vs. Ag|Ag^+. The resistance of the solution R_{SOL} was subtracted for all temperatures and samples for the convenience of R_{CT} comparison.

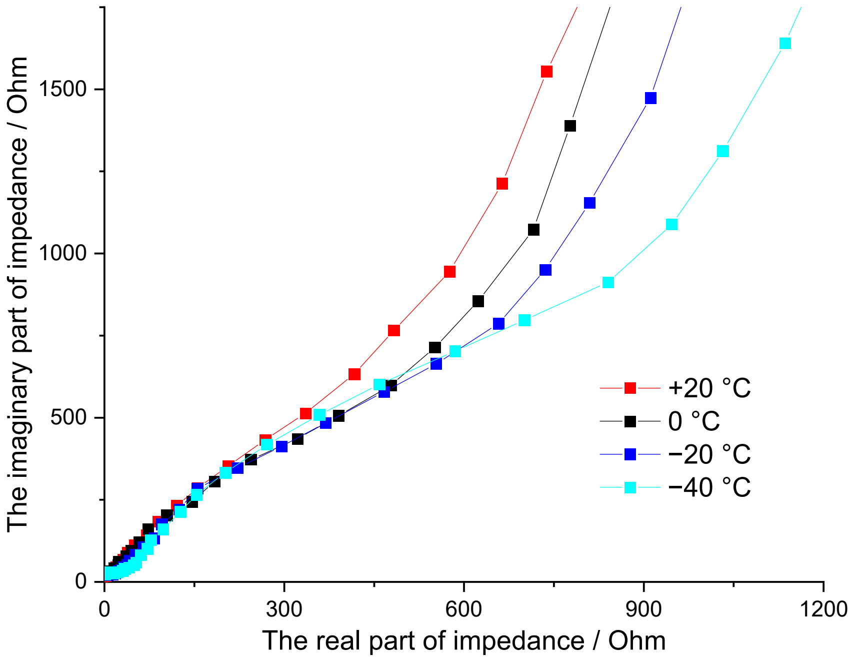

Fig.S7. Examples of impedance spectra of poly[Ni(CH_3Salen)] film recorded in Me_4NBF_4 /AN solution at different temperatures at 0.190 V vs. Ag|Ag^+. The resistance of the solution R_{SOL} was subtracted for all samples for the convenience of R_{CT} comparison.

Fig.S6 demonstrate EIS spectra of the sample electrodeposited and tested in 0.1M Me_4NBF_4/AN solution (the one with small pores). One can see that charge transfer related resistance R_{CT} of the sample is strongly dependent on temperature. R_{CT} grows and order of magnitude with the 60°C decrease in temperature. Fig.S7 demonstrates EIS spectra of the other sample (electrodeposited and tested in 0.1M Bu_4NBF_4 /AN solution, the one with big pores). One can see that charge transfer resistance of this sample demonstrates rather moderate dependence on the temperature. R_{CT} grows ~2 times with the 60°C decrease in temperature.

1s) Determination of the diffusion coefficients for charge transfer through homo-, bilayered- and co-polymers of 3-methyl- thiophene and N-methylpyrrole Int. J. Electrochem. Sci., 7 (2012) 785 – 805

2s) Apparent diffusion coefficient of intercalated species measured with PITT A simple formulation doi:10.1016/j.electacta.2005.08.046

3s) Aurbach, D., Levi, M.D., Lev, O. et al. Behavior of lithiated graphite electrodes comprising silica based binder. Journal of Applied Electrochemistry 28, 1051–1059 (1998). https://doi.org/10.1023/A:1003412917632

4s) Cabanel, R., Barral, G., Diard, J.P. et al. Determination of the diffusion coefficient of an inserted species by impedance spectroscopy: application to the H/H x Nb2O5 system. J Appl Electrochem 23, 93–97 (1993). https://doi.org/10.1007/BF00246943

5s) Theory of the electrochemical impedance of anomalous diffusion https://doi.org/10.1016/S0022-0728(00)00497-6

6s) New approach of electrochemical systems dynamics in the time domain under small-signal conditions: III – Discrimination between nine candidate models for analysis of PITT experimental data from LixCoO2 film electrodes https://doi.org/10.1016/j.jelechem.2009.01.012
